# Production and optimization of novel Sphorolipids from *Candida parapsilosis* grown on potato peel and frying oil wastes and their adverse effect on Mucorales fungal strains

**DOI:** 10.1186/s12934-023-02088-0

**Published:** 2023-04-25

**Authors:** Amr S. Al-kashef, Mohamed U. Nooman, Mona M. Rashad, Amr H. Hashem, Mohamed Abdelraof

**Affiliations:** 1 Biochemistry Department, Biotechnology Research Institute, National Research Centre, Cairo, 12622 Dokki Egypt; 2 Microbial Chemistry Department, Biotechnology Research Institute, National Research Centre, Cairo, 12622 Dokki Egypt; 3 Botany and Microbiology Department, Faculty of Science, Al-Azhar University, Cairo, 11884 Egypt

**Keywords:** Production, Sophorolipids, Optimization, *Candida parapsilosis*, Potato peel waste, Frying oil waste, Mucorales

## Abstract

**Brief introduction:**

Mucormycosis disease, which has recently expanded with the Covid 19 pandemic in many countries, endangers patients' lives, and treatment with common drugs is fraught with unfavorable side effects.

**Aim and objectives:**

This study deals with the economic production of sophorolipids (SLs) from different eight fungal isolates strains utilizing potato peels waste (PPW) and frying oil waste (FOW). Then investigate their effect against mucormycetes fungi.

**Results:**

The screening of the isolates for SLs production revealed the highest yield (39 g/100 g substrate) with most efficiency was related to a yeast that have been identified genetically as *Candida parapsilosis*. Moreover, the characterizations studies of the produced SLs by FTIR, ^1^H NMR and LC–MS/MS proved the existence of both acidic and lactonic forms, while their surface activity was confirmed by the surface tension (ST) assessment. The SLs production was optimized utilizing Box-Behnken design resulting in the amelioration of yield by 30% (55.3 g/100 g substrate) and ST by 20.8% (38mN/m) with constant level of the critical micelle concentration (CMC) at 125 mg/L. The studies also revealed the high affinity toward soybean oil (E_24_ = 50%), in addition to maintaining the emulsions stability against broad range of pH (4–10) and temperature (10–100℃). Furthermore, the antifungal activity against *Mucor racemosus, Rhizopus microsporus,* and *Syncephalastrum racemosum* proved a high inhibition efficiency of the produced SLs.

**Conclusion:**

The findings demonstrated the potential application of the SLs produced economically from agricultural waste as an effective and safer alternative for the treatment of infection caused by black fungus.

## Introduction

Black fungus infection or mucormycosis is a rare disease caused by mucormycetes fungi; mostly it is due to the invasion of the genera *Mucor* and *Rhizopus*. Because they are fast-growing fungi, the infection spread rapidly and patient's life become in a great risk when mucormycosis spreads from the nose to the eye and then the brain. Mucormycetes fungi are described as opportunistic fungi which infect the immunocompromised patients. Therefore, the most common high-risk cases are diabetics, renal disease and cancer patients and recently, the post-acute coronavirus (COVID-19) syndrome due to, low white blood cell counts, and long-term immunosuppressive steroid therapy [1, 2]. Mucormycosis mortality rate is 54%, according to the United States Centers for Disease Control and Prevention reports [3]. Hence, when the infection has developed to the level of the eye, the eyes must be removed immediately. Yet, more than 80% of the patients need such a surgery to stop the deterioration of the case as it prevents the proliferation of the fungi to the brain [3].

Mucormycosis are usually treated with amphotericin B, for at least 4–6 weeks, other antifungal agents may be used such as isavuconazonium sulfate and posaconazole especially for those with a compromised immune system. The problem related to these drugs is in fact due to their undesirable long list side effects, including nausea, vomiting, headache, fever, chills, irregular heartbeat, muscle cramps or pain, abnormal liver blood tests, low blood potassium, low blood pressure and shortness of breath [2, 4].

Therefore, natural compounds have received a lot of attention especially those have the same pharmacological effects as synthetic drugs, and fewer side effects. Most of the natural compounds are characterized by their safety for human consumption and their environmental friendliness. The SL compounds, as a member of biosurfactants family, are an example of these natural compounds that dissolve in water as well as oil, which provide them a unique properties and a wide range of applications [5, 6].

The biggest obstacle to SLs' widespread distribution, despite their advantages over synthetic surfactants, is their high manufacturing cost. Recent studies therefore, concentrated on several axes: The first is connected to the raw material, which accounts about 10% to 50% of the entire cost, and thus employing agro-industrial wastes as a substrate for the manufacturing process would be cost-effective, in addition to eliminating the negative environmental effects of such residues [7]. Another concept that may reduce the production cost is the type of fermentation. Generally, solid state fermentation (SSF) considered being an effective and economic process for the production, due to the low consumption of water, lower energy needed, higher productivity and more stability for the product [5, 8, 9].

Sophorolipids in many reports have been mentioned to have anti-cancer, anti-hypercholesteremic, anti-corrosion, anti-food spoilage, antimicrobial and bioremediation effects [5, 6, 10–12]. A few reports on the production of natural compounds from *Candida parapsilosis,* have been published where, Garg and Chatterjee [13] isolated docosenamide as a type of biosurfactant.

The aim of this study was to employ the isolated yeast strain (*Candida parapsilosis*) for the first time in the production and optimization of SLs utilizing a mixture of two different agro-industrial wastes namely PPW and FOW. In addition to investigate the effect of the newly produced SLs for the first time as antifungal agent against black fungi (mucormycosis).

## Materials and methods

### Materials

The PPW and FOW were collected from the remnants of some fried potato processing factories. Mucorales fungal strains, *Rhizopus microsporus* (Accession no. MK623262), *Mucor racemosus* (Accession no. MG5475711) and *Syncephalastrum racemosum* (Accession no. MK621186) were kindly supplied by Mycology culture collection, Plant and Microbiology Dept., Faculty of science, Al-Azhar University. Egypt. All chemicals and solvents used through this study were of analytical grade.

### Yeast isolation and preservation

Yeast strains were isolated from the surrounding area of chemical pollutants environment, Cairo, Egypt. In this regard, Potato Dextrose Agar (PDA) medium with 200 mg L^−1^ chloramphenicol was prepared and poured into the sterilized Petri dishes after sterilization. Then, the soil and sludge samples were serially diluted in sterilized bi-distilled water according to a conventional dilution methodology until a dilution of 10^–7^ was reached. Subsequently, dilutions ranging from 10^–5^ to 10^–7^ were vortexed, transferred and spread onto the solidified medium with 100 µl and incubated at 28 °C for 5 days. During this time, all developed yeast cultures were picked up and kept at 4 °C in PDA medium slants for further studies [14]**.**

### Preparation the culture medium

Initially, each yeast isolate was pre-activated by cultivation on Potato Dextrose Broth (PDB) for 24 h at 28 °C in a shaking environment (Incubated/Refrigerated Orbital Shaker, Thermo SCIENTIFIC- USA). Screening of different yeast isolates for their ability to produce biosurfactant was investigated using a low-cost culture medium that based on PPW and FOW. For this purpose, PPW (5 g/l) and FOW (5 g/l) that had been prepared and moistened (60%) with the Czapek Dox medium (g/l): NaNO_3_, 2; K_2_HPO_4_,1.0; MgSO_4_.7H_2_O, 0.5; KCl, 0.5; and FeSO_4_, 0.001, along with adjustment of pH at 7.2. Under SSF conditions, PPW and FOW were used as carbon and inducer sources for SL synthesis. The sterilized PPW-FOW was inoculated with 1.0 × 10^7^ cfu/ml of yeast cells and incubated for 5 days at 28 °C under a static condition.

### Extraction of SLs

#### Extraction by methanol

The SLs were isolated according to a modified method of [5]**,** whereas 100 ml of methanol was added to the cultures, then the mixture was shaken at 160 rpm for 2 h at 30 ℃ with a reciprocal shaker (Incubated/Refrigerated Orbital Shaker, Thermo SCIENTIFIC- USA). Then through Whatman No. 2 filter paper, the extract was filtered. The methanol was evaporated at 40 ℃ by a rotary evaporator (heidolph cooling analog vacuum controller G1-Germany) to obtain the methanol SL extract.

#### Re-extraction by ethyl acetate

Ethyl acetate (100 ml) was added to the cultures remained after the methanol extraction, then the mixture was shaken at 180 rpm for 2 h at 40 ℃ with a reciprocal shaker. Whatman No. 2 filter paper then, used to filtrate the extract. The solvent was then evaporated at 40 ℃ by the rotary evaporator to obtain the ethyl acetate SL extract [6].

### Qualitative screening of produced SLs by oil displacement method

The oil displacement test was carried out by dropping 15 µl of motor oil onto the surface of a 40 ml distilled water layer housed in a Petri dish (15 cm in diameter) that spread all over the water surface area. Following that, 10 µl of the SLs extracts from different yeast isolates were applied to the oil surface. The average diameters of the clear zones in triplicate trials were measured and recorded, then the percentage of the clear zone related to Petri dish diameter was estimated [15]. The robust yeast strain from 8 isolated strains, has the ability to produce the highest activity oil displacement and highest productivity, was then further identified at the molecular level.

### Molecular identification of isolated strains

The most efficient SL-yeast producer was sequentially subjected to the molecular identification using 18S rDNA based molecular approach. Isolation of total genomic DNA, purification from any primer dimer, and sequencing of the targeted gene after PCR amplification was carried out according to a standard method of Macrogen Company (Seoul, South Korea; https://www.macrogen.com). Universal primers for fungal strains, NS1 “GTAGTCATATGCTTGTCTC” and NS8 “TCCGCAGGTTCACCTACGGA” were used in the amplification process. PCR protocol was applied as the following conditions: initial denaturation at 94 °C for 5 min followed by 40 cycles of denaturation at 92 °C for 30 s, annealing at 54 °C for 45 s and an extension step at 72 °C for 5 min. The amplicons were purified and then directly sequenced using the ABI 3730 DNA Analyzer (Applied Biosystems). The resulting sequence were aligned and compared with the related sequences deposited in GenBank (http://blast.ncbi.nlm.nih.gov/Blast.cgi) using the Basic Local Alignment Search Tool (BLAST). Consequently, phylogenetic analysis of the obtained sequence was conducted using Molecular Evolutionary Genetic Analysis Software (MEGA version X) and the phylogenetic tree with the related strains was then constructed. The 18S rDNA gene sequence of the yeast strain used in this study have been deposited in the GenBank nucleotide sequence database under the accession number MT860358, and the strain was identified as *Candida parapsilosis.*

### Surface tension (ST) measurement and critical micelle concentration (CMC)

The ST of the prepared SL (0.2% aqueous solution) was estimated at room temperature using KrÜss Processor tensiometer-K100, Germany (applying the ring method). Each concentration was repeated three times and the means were reported. The CMC was determined from the break point in ST versus concentrations of the prepared SL [16]**.**

### Optimization process and statistical analysis

A study regarded with the enhancement of the SL production by *Candida parapsilosis* was then carried out via One-Factor-at-a-Time (*OFAT*) procedure. Therefore, different cultural conditions were optimized under SSF technique in order to determine the significant ones. In this way, the primary factors impacting SL production was identified by investigating only one parameter per test while, the other variables are kept constant. Accordingly, the insignificant factors were spontaneously neglected, and the significant factors including, FOW concentration, moisture content, and initial pH were investigated in the next experiment for further production improvement. For this purpose, further optimization of the significant parameters that able to increase the SL activity, Box-Behnken design (BBD) was created. The BBD is a mathematical approach for deeply identifying the critical factors that influence the response [17]**.** In the current study, three factors were selected for decreasing ST using BBD as mentioned in (Table 1).

**Table 1:** The selected factors and levels for optimization process

Factor	Low level (−)	High level ( +)
FOW concentration	5	10
Initial pH	4	6
Moisture content	40	60

The experimental design composed of 15 experimental runs was created; among these, three run was carried out at the center point values, while each remaining runs will apply at 2-levels by combinations of high ( +) and low ( −) levels of all parameters. In the BBD, two levels were utilized to determine whether the maximum production was obtained at lower or higher concentration of the variables by comparing them with the experimental results obtained from center point values [18]. The significance of the model was determined by analysis of variance, the regression equation was obtained, a P value less than 0.05 indicates that the model term is significant.

### Validation of the results

Following the theoretical optimization of the three independent parameters for reducing CMC value by *Candida parapsilosis* SL, the response optimizer's optimum conditions were experimentally applied and compared to the expected outputs. The applications of the conditions were carried out in three replicates and the results were reported as mean ± standard deviation.

### Structural characterization of isolated SLs

#### Fourier transform infrared spectroscopy (FTIR)

Attenuated total reflectance (ATR)-FTIR assessment were performed on a Bruker VERTEX 80 (Germany) combined Platinum Diamond ATR, using of a diamond disc as an internal reflector at a range of 4000–400 cm^-1^ with a resolution of 4 cm^-1^ and a refractive index of 2.4.

#### ^1^H NMR spectra analysis

A Varian Mercury VX-300 NMR spectrometer was used to record the NMR spectra. In deuterated chloroform (CDCl_3_), ^1^H spectra were conducted at 300 MHz. Chemical shifts are quoted in and are related to solvent shifts.

#### LC–MS/MS

The produced biosurfactants were analyzed by liquid chromatography–electrospray ionization–tandem mass spectrometry (LC–ESI–MS/MS). ExionLC AC system and SCIEX Triple Quad 5500 + MS/MS system used for separation, and the electrospray ionization (ESI) for detection. However, separation was carried out utilizing a Ascentis® C18 Column with the dimensions of 4.6 × 150 mm, 3 µm. The mobile phases were represented by two eluents, where A was: 0.1% formic acid and B: 0.1% formic acid in acetonitrile (LC grade)**.** The program for the mobile phase was as follows: 10% B at 0–2 min, 10–90% B from 2 to 30 min, 90% B from 30 to 36 min, 10% at 36.1, 10% from 36.1 to 40 min. For MS/MS analysis, the flow rate was 0.7 ml/min and the volume of injection was 10 µl. The positive ionization mode was performed with a scan (EMS-IDA-EPI) from 100 to 1000 Da for MS1 using the following parameters: curtain gas: 25 psi; 5500 IonSpray voltage; 500 °C as a temperature source; ion source gas 1 and 2 were 45 psi and 50–1000 Da for MS2 with a declustering potential: 80; collision energy: 35; collision energy spread: 20. Compounds’ identification was performed using MS-DIAL software version 4.70 and FiehnHILIC library.

### Emulsification studies of the prepared SLs

According to the estimated ST (48 mN/m) in a concentration of 50 mg/l, 2 ml of SL solutions were prepared in distilled water at room temperature for each emulsification index (E_24_) and the emulsion stability test. Where 2 ml of SL mixed with 2 ml of hydrocarbons (motor oil and hexane) or vegetable oils (corn and soybean oils) in a graduated screw cap test tube, then the mixture was shacked vigorously by vortex mixer (heidolph Reax top) at high speed for 2 min. The height of the emulsion layer was measured and divided by the total mixture height then multiplied by 100 to express the emulsification index values. While, the emulsion stability was determined after 24 h, then observed in 7 days intervals [19]**.**

### Stability studies against extreme environmental conditions

Stability studies were carried out for SLs extract using soybean oil as follows. The temperature stability was examined by heating 2 ml of 50 mg/l in distilled water at 0, 10, 25, 55, 70 and 100 °C for 15 min then left to settle down to room temperature, after which the emulsification index was measured. As for the stability of pH, 50 mg/l of SLs extracts were prepared at different pH values (2, 4, 6, 8 and 10) using HCl or NaOH and then emulsification activities were assessed. The effect of salinity concentrations (2, 4, 6, 8 and 10 NaCl %) was also measured [20].

### Anti-Mucorales activity of produced SLs:

Using the agar well diffusion method, the effectiveness of produced SL in preventing the proliferation of Mucorales strains was tested against *Rhizopus microsporus* (Accession No. MK623262), *Mucor racemosus* (Accession no. MG5475711), and *Syncephalastrum racemosum* (Accession No. MK621186) [21]. Pre-activation of Mucorales strains was carried out using PDB medium for 48 h., which the inoculum concentration was adjust to 10^6^/mL, approximately. The anti-Mucorales activity of methanol extract SL was compared to fluconazole and Amphotericin B, two commonly used antifungal drugs. The inhibition zone diameter (mm) was used to measure SLs ability to prevent fungal proliferation [22, 23]. Furthermore, assessed of the SLs minimum inhibition concentration (MIC) against each Mucorales strain using the broth micro dilution method was also investigation according to CLSI protocol. To prepare the stock solution, 100 mg of SL was dissolved in 1 ml of methanol and the reference drugs (Fluconazole, and Amphotericin B) were also prepared in order to get the desired concentrations. Dilutions of SL with PDB were performed to obtain known concentrations from 25 to 400 µg/ml. In comparison to the standard drugs, each investigated strain was incubated with the examined SL for 48 h at 28 °C. The PDB mixed with methanol was displayed as the control growth samples. After that, each treated sample was inoculated with 50 µl to PDA plate and incubated for 72 h at 28 °C. Determination of MIC value for both SL, and reference drugs was identified by the lowest concentration of each compound that yield a reduced number of colonies forming unit (CFU) comparing to the untreated samples [22, 24].

### Statistical analysis

The results are reported as Mean ± Standard error (S.E.) for experiments repeated at least three times.

## Results and discussions

### Screening and production of SLs

**Fig. 1:**
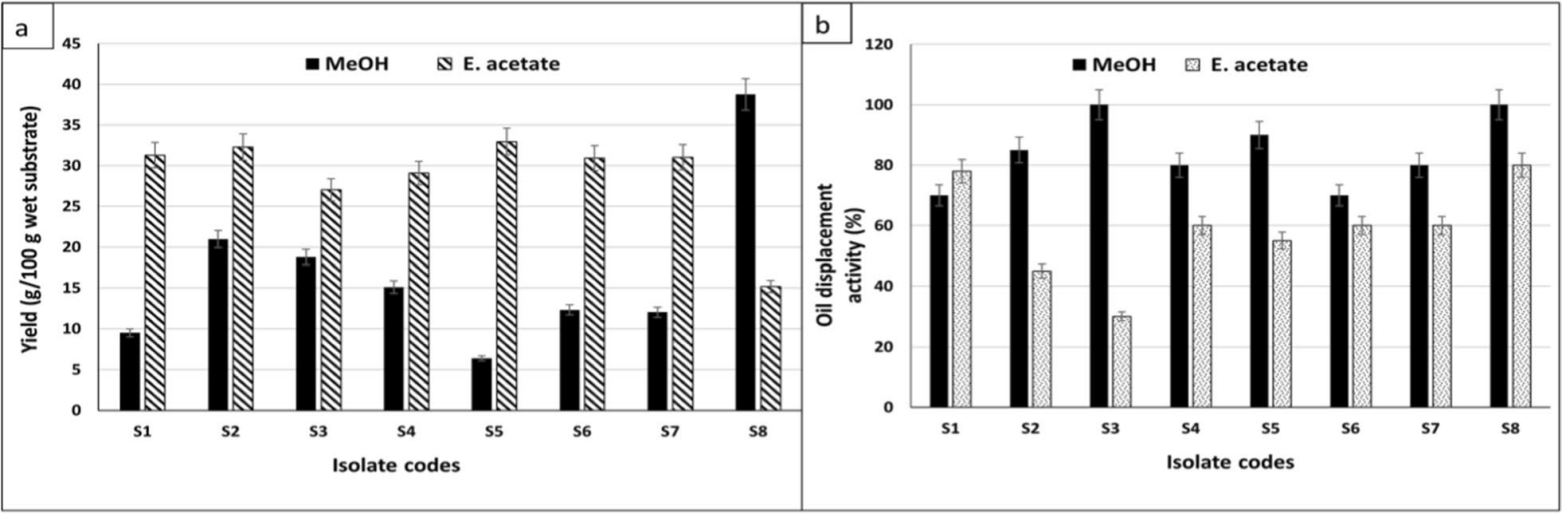
Screening of both produced SLs methanol and ethyl acetate extracts productivity (**a**) and activity (**b**). Data were expressed as mean ± S.E. of 3 experiments

The capability of yeast isolates to grow on solid state medium containing PPW and FOW as carbon sources and inducer agents, respectively, actually encouraged SLs biosynthesis. Among the eight isolates, one isolate (S8) displayed the highest oil displacement activity of the produced SLs (100 and 80%) for both methanol and ethyl acetate extracts, respectively (Fig. 1a).

This was followed by isolate coded as S3 (100 and 30%) for both extracts, while S5 gave 90 and 55% also for both extracts, respectively. In addition, the isolate coded as S1 gave the highest activity (78%) for the ethyl acetate extract only. On the other hand, the methanol extract of isolate S8, gave the highest yield (39 g/100 g substrate) followed by S2 isolate methanol extract (21 g/100 g substrate). While, S5 and S2 isolates showed the highest yield for ethyl acetate extracts (33 and 32 g/100 g substrate, respectively). These results indicated the superiority of isolate S8 during the screening test, whereas the S8 methanol extract, achieved the highest productivity associated with the highest activity. Therefore, the encoded isolate S8 was chosen for further studies in this investigation (Fig. 1b).

### Identification of S8 isolate by molecular characterization:

**Fig. 2:**
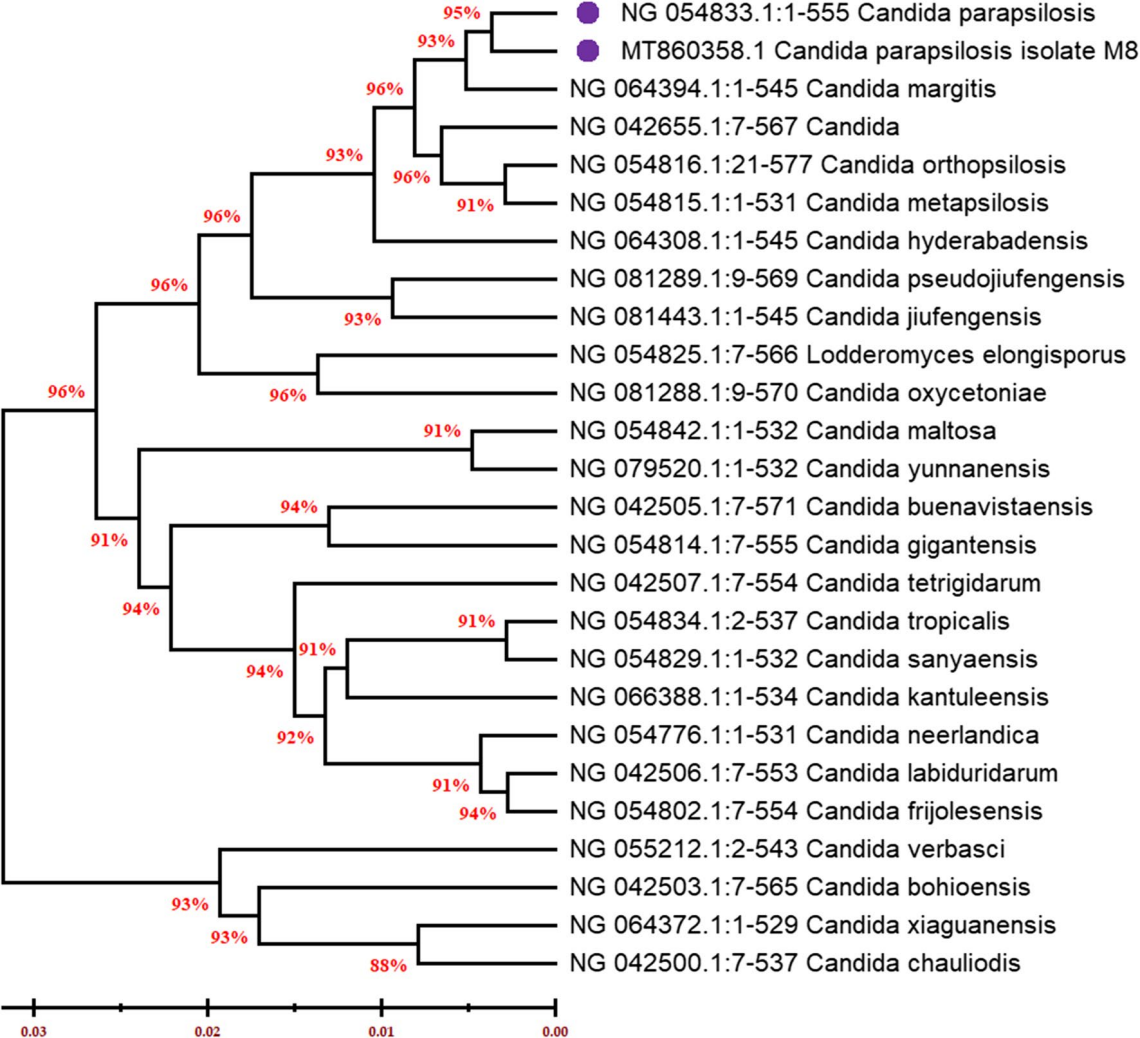
Phylogenetic tree of the SL-producing isolate

Isolation of total genomic DNA from the most potent SL-producing isolate (S8) was performed to complete the identification study based on a wide molecular characterization. Following that, PCR amplification of the targeted gene 18S rRNA was carried out using universal primers and the PCR product was purified and finally sequenced. The resulting sequence was then analyzed using BLAST in GenBank (http://blast.ncbi.nlm.nih.gov/Blast.cgi). Relatedness of the almost complete targeted sequence of 18S rRNA gene was found to be corresponding to *Candida* species, similarity with more than 98%. Moreover, the phylogenetic analysis showed that the isolate was more related with *Candida parapsilosis* strain (Fig. 2). Accordingly, the microscopic observations and molecular characterizations suggested that this isolate S8 was closely related to *Candida parapsilosis* strain and deposited as *Candida parapsilosis* S8 in GenBank under accession number, MT860358.

### Statistical optimization analysis of SLs production medium

Recently, there has been an urgent need for the sustainable production of SLs using low-cost culture medium based on agro-industrial wastes. Commonly, these wastes were accumulated in the environment causing severe pollution like agricultural peels, and used frying oils [25, 26]. Utilization of waste as the sustainable substrate is not only preferred for improving the profitability of the process but also helpful in the significant management of the waste that is being a source of environmental contamination. Furthermore, optimization is another concept to maximize the profit as it aims to increase the production level of SLs [27, 28]**.** The production and statistical optimization of SLs by *C. parapsilosis* under SSF has been implemented in four sequential steps; BBD experimental design, doing the experiment, data analysis and validation of the results. Prior to statistical modeling, the different factors as mentioned in the materials and methods section were examined in order to select the significant ones that influence the production process and determine the optimum maximum and minimum levels based on OFAT method. Accordingly, from OFAT approach, the most impacted factors on the culture medium under SSF were initially selected. In which, these factors could be notably enhance the activity and yield of produced SLs resulting in decreasing the ST value among other factors (Data not shown). Further optimization studies would be conducted via modeling of SLs production using BBD to enhance the ability to reduce the ST level and increase the SL production yield. In the current study, BBD with 15 runs was applied for SLs production (decreasing ST value) by *C. parapsilosis* as shown in (Table 2).

**Table 2:** Analysis of variance

Source	DF	Adj SS	Adj MS	F-value	p-value
Model	9	554.850	61.650	8.87	0.014
Linear	3	302.250	100.750	14.50	0.007
pH	1	153.125	153.125	22.03	0.005
Moisture content	1	21.125	21.125	3.04	0.142
FOW	1	128.000	128.000	18.42	0.008
Square	3	248.100	82.700	11.90	0.010
pH*pH	1	83.308	83.308	11.99	0.018
Moisture content*moisture content	1	0.231	0.231	0.03	0.863
FOW*FOW	1	180.923	180.923	26.03	0.004
2-Way Interaction	3	4.500	1.500	0.22	0.882
pH*Moisture content	1	4.000	4.000	0.58	0.482
pH*FOW	1	0.250	0.250	0.04	0.857
Moisture content*FOW	1	0.250	0.250	0.04	0.857
Error	5	34.750	6.950		
Lack-of-Fit	3	34.750	11.583	*	*
Pure Error	2	0.000	0.000		
Total	14	589.600			

R-sq (94.11%), R-sq(adj) (83.50%), R-sq(pred) (5.70%)

**Fig. 3:**
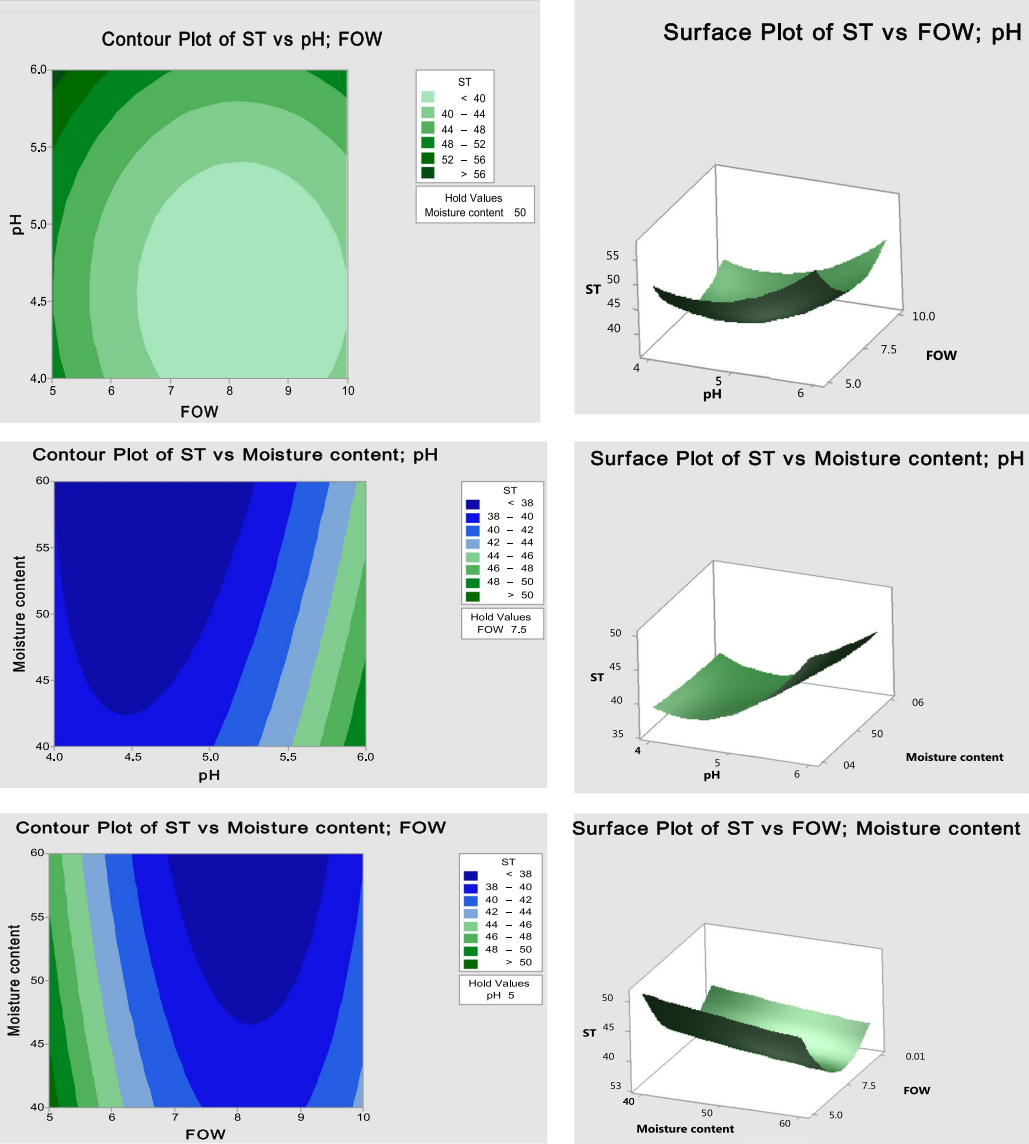
Response surface plot and contour line plot of the effect of cross-interaction among different variables on SL production. This Figure show 2-way interactions between each two factors at different levels: **1** PH*FOW, **2** Moisture content*PH, **3** Moisture content*FOW

The F value (Fisher’s statistical analysis) and P-value (> 0.0001) were used for determining the significance of the model. Low values of P indicate the high significance of the corresponding coefficient whereas large t and F values indicate the significance of corresponding coefficients [29]**.** ANOVA results showed that, the model is highly significant where P value was 0.014. Moreover, model terms initial pH, and FOW were significant factors for SL production where P-value was 0.005 and 0.008 respectively (Table 3, Fig. 3). Also, two way interactions between each two factors was carried out through BBD, where FOW*FOW was the significant (0.004) among other interactions. Furthermore, the Model F-value is 8.87 which emphasizing the model is significant. Besides, the "Pred R-Squared" of 94.11% is reasonable agreement with all the "Adj R-Squared" of 83.5%.

**Table 3:** BBD for improvement production of SL

Run no	Initial pH (*X*_*1*_)	Moisture content (*X*_*2*_)	FOW (*X*_*3*_)	ST (mN/m)	
				Observed	Predicted
1	5	50	7.5	38	38.000
2	5	50	7.5	38	38.000
3	6	50	5.0	60	57.875
4	6	40	7.5	49	50.000
5	4	50	5.0	48	49.625
6	6	60	7.5	42	44.750
7	5	40	5.0	50	51.125
8	5	60	10.0	41	39.875
9	4	50	10.0	39	41.125
10	5	40	10.0	42	42.625
11	6	50	10.0	52	50.375
12	5	50	7.5	38	38.000
13	4	40	7.5	42	39.250
14	5	60	5.0	48	47.375
15	4	60	7.5	39	38.000

The initial order model equation created by BBD showed the dependence of SL production on the medium constituents regression equation as follows:

$$ \begin{aligned}   {\text{ST}}\left( {{\mathbf{mN}}/{\mathbf{m}}} \right) = {2}0{6}.{7} - {38}.{9} X_{1} \, + \,0.0{1} X_{2} - {19}.{4}0 {X_{3}}_{ + } {4}.{75} X_{1} *X_{1} \, + \,0.00{25} X_{2} *X_{2} \, + \,{1}.{12}0 X_{3} *X_{3} - \,  0.{1}00 X_{1} *X_{2} \, + \,0.{1}00 X_{1} *X_{3} \, + \,0.0{1}00 X_{2} *X_{3} .   \end{aligned} $$

### Validation of the results

**Fig. 4:**
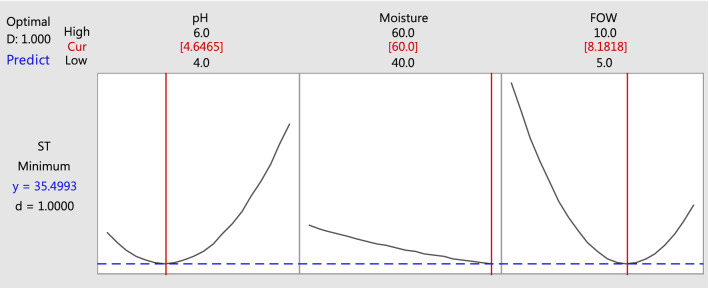
Response optimizer for SL production (decreasing of ST) by* C. parapsilosis*

The numerical optimization of the used variables in the SL production by *Candida parapsilosis* resulted in three different conditions with desirability of (1.0) and minimum ST value (Fig. 4).

After testing these optimized values with three trails, the total decreasing of the predicted ST values was found to be near with the practical result (38 mN/m). The agreement of the predicted results of the models with the practical results indicates the validity of the designated model. The validation of the obtained results showed average confidence of 90.71% and minimum ST value (35.4993 mN/m). The optimum conditions of oil content were 8.1818 g/L; pH 4.6465 and moisture content was 60% for the predicted at 35.4993 mN/m, which close to that obtained from the practical study at 38 mN/m.

The supplementation of the *C. parapsilosis* culture medium with the FOW as indicator agent had a significant effect on SL production more than other oils such as coconut oil, olive oil, and soybean oil and that probably related with the higher free fatty acids content in waste oil [30, 31]**.** Furthermore, the addition of FOW with a known concentration was found statistically enhancing the SL formation by *Candida tropicalis* [31]. In consistence with our study, biosurfactant production by *Bacillus pumilus* was not related directly with the FOW concentration in the culture medium, which any FOW concentration could cause induction of biosurfactant synthesis [32].

### Functional characterization of *C. parapsilosis* SLs by ST and CMC index

**Fig. 5:**
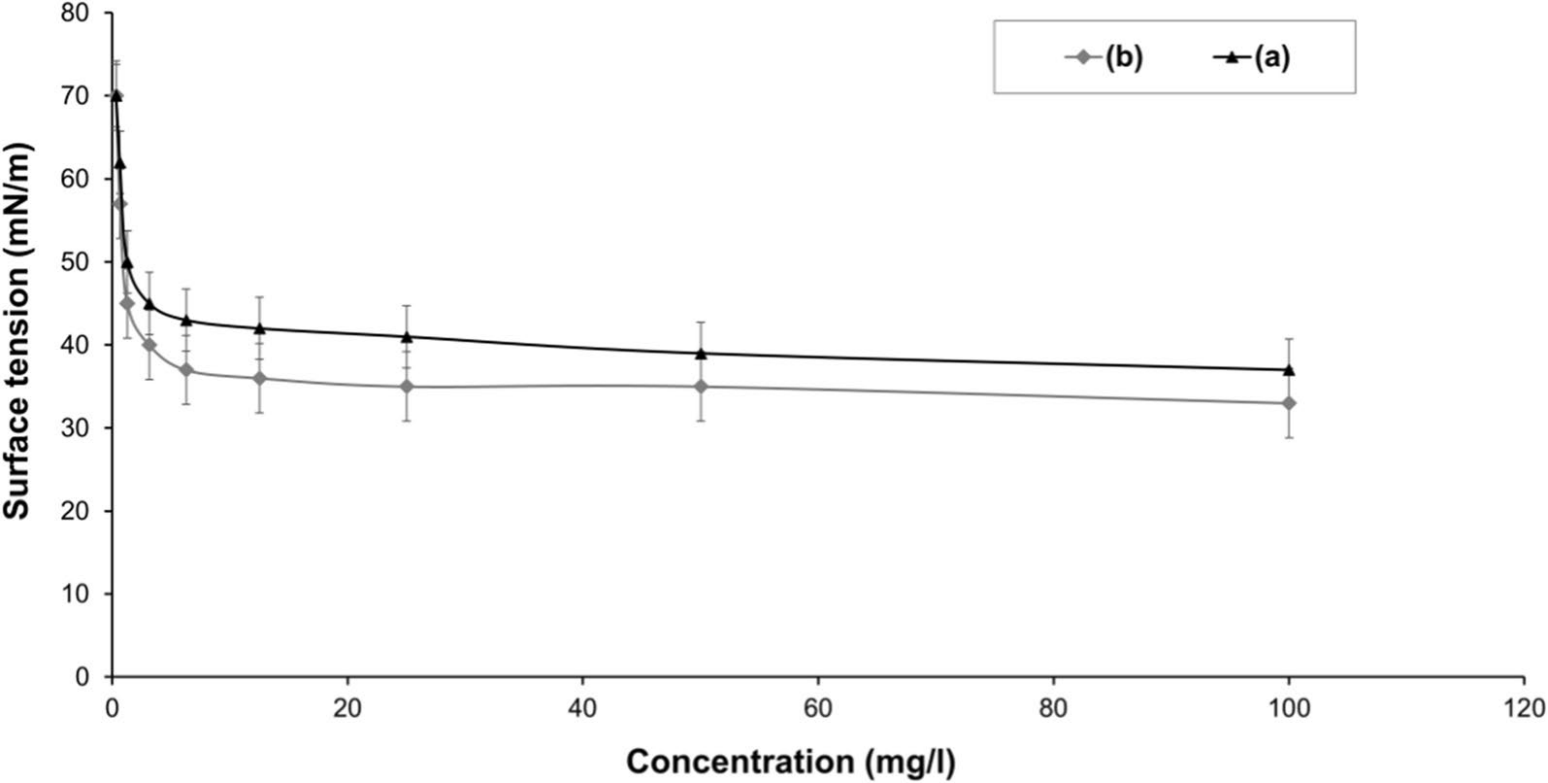
CMC and minimum ST of the isolated SL before optimization (**a**) and after optimization (**b**). Data were expressed as mean ± S.E. of 3 experiments

CMC index is widely used to evaluate surface activity therefore, CMC values are important to determine the potential applications of biosurfactants. Furthermore, CMC is significant parameter to determine whether the compound is economical related to its productivity. The ST at CMC level was estimated for the *C. parapsilosis* SLs methanol extract during the optimization process. The methanol fraction (SLs) exhibited a reduction in water ST from 70 mN/m to 48 mN/m at a CMC level of 125 mg/l, before the optimization, while the results showed an improvement of the reduction of ST to 38 mN/m at the same CMC concentration (Fig. 5).

Rashad et al. [6], mentioned a similar finding for the reduction of the ST exerted by SL extracted with methanol from *C. bombicola* grown on sunflower oil cake which was 45 mN/m. Later, [5] used motor oil waste as substrate to grow *C. bombicola* in which they found that SL methanol fraction exhibited a good ST (37.6 mN/m) at the CMC of 80.51 mg/l. In fact, the CMC value of the produced SL in this investigation was higher than that observed by [5]**.** On the other hand, [33] illustrated that SL isolated from the *Rhodotorula babjevae* grown on Bushnell-Haas medium had a higher efficiency to reduce the ST of water (32.6 mN/m) and similar CMC level of 130 mg/l. While, SL isolated from novel yeast strain *Metschnikowia churdharensis* showed also a lower ST (35 mN/m) with higher CMC value (5 g/l) compared to results obtained in this investigation [34].

### Molecular characterization of *C. parapsilosis* SLs

#### Fourier transform infrared spectroscopy (FTIR):

**Fig. 6:**
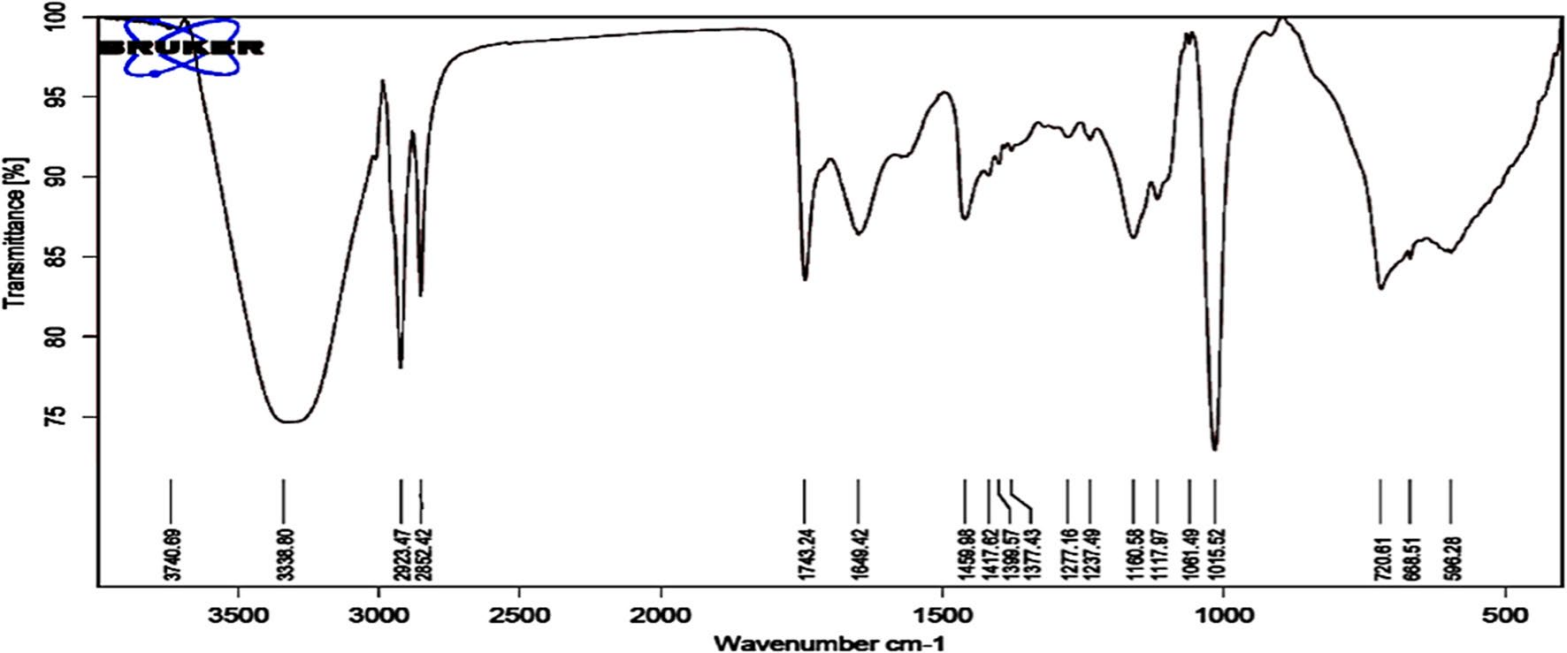
FTIR spectra of SL produced by *C. parapsilosis*

The isolated methanol SLs extract was identified and characterized by FTIR as shown in (Fig. 6).

Data revealed the presence of broad band at 3388 cm^−1^ which is corresponding to the O–H stretch in SLs extract structure. Asymmetrical stretching (V_as_ CH_2_) and symmetrical stretching (V_s_ CH_2_) of methylene groups were observed in 2923 and 2852 cm^−1^. The C = O stretching from lactone ester or acids appeared in the absorption band at 1743 cm^−1^. The band at 1459 cm^−1^ was actually corresponded to the C–O–H in plane binding of carboxylic acid group (–COOH). Absorption at 1237 cm^−1^ was corresponded to C = O band from acetyl esters, while the stretch of the C-O band of C (-O)-OC in lactones exists was observed at 1160 cm^−1^. Sophorose moiety however, was observed at 1061 cm^−1^ in the C–O stretch of C–O–H group. The IR spectra also detected the absorption band at 720 cm^−1^ for C = C in the SL structure. These two bands at 1459 and 1743 cm^− 1^ were found to be typical with SLs in literature reported by [5, 6] and [33], confirming that the tested SLs methanol fraction was a mixture of both lactone and acidic forms.

#### ^1^H NMR spectra analysis

The structure of the produced SLs (methanol extract) was elucidated using ^**1**^H NMR analysis. The spectra signals indicated the existence of glycolipid-type structure of biosurfactant, where signals of the vinyl group (–CH = CH–) were at 5.31≈5.33 ppm. Also, signal at 2.03 ppm appeared as evidence for the presence of –CH3 group. The occurrence of fatty acid chain moiety shown by multiple peaks from 1.22 to 1.27 ppm, while glucose molecules signals (sophorose) were resonating at 3.44 and 3.58 ppm. The signals at 4.92 ppm put the accent on the existence of lactone form. According to the results of the analyses by FT-IR and ^1^H NMR, the extracted structure confirmed to be SLs compound in the form of acid and lactone.

The signals obtained were similar to the ^**1**^H NMR spectra of the SL compounds reported by other researchers [12, 16, 35–37].

On the other side, [13] were the first to isolate biosurfactant from *C. parapsilosis* and they classified the product as docosenamide, by acid precipitation as a method for extraction. It is well known, that *Candida sp*. have the ability to synthesize sophorolipids under the appropriate conditions related to the type of substrate and fermentation conditions [9, 10, 12, 16]. In fact, our study was the first to confirm the isolation of SLs from *C. parapsilosis.*

#### LC–MS/MS

**Fig. 7:**
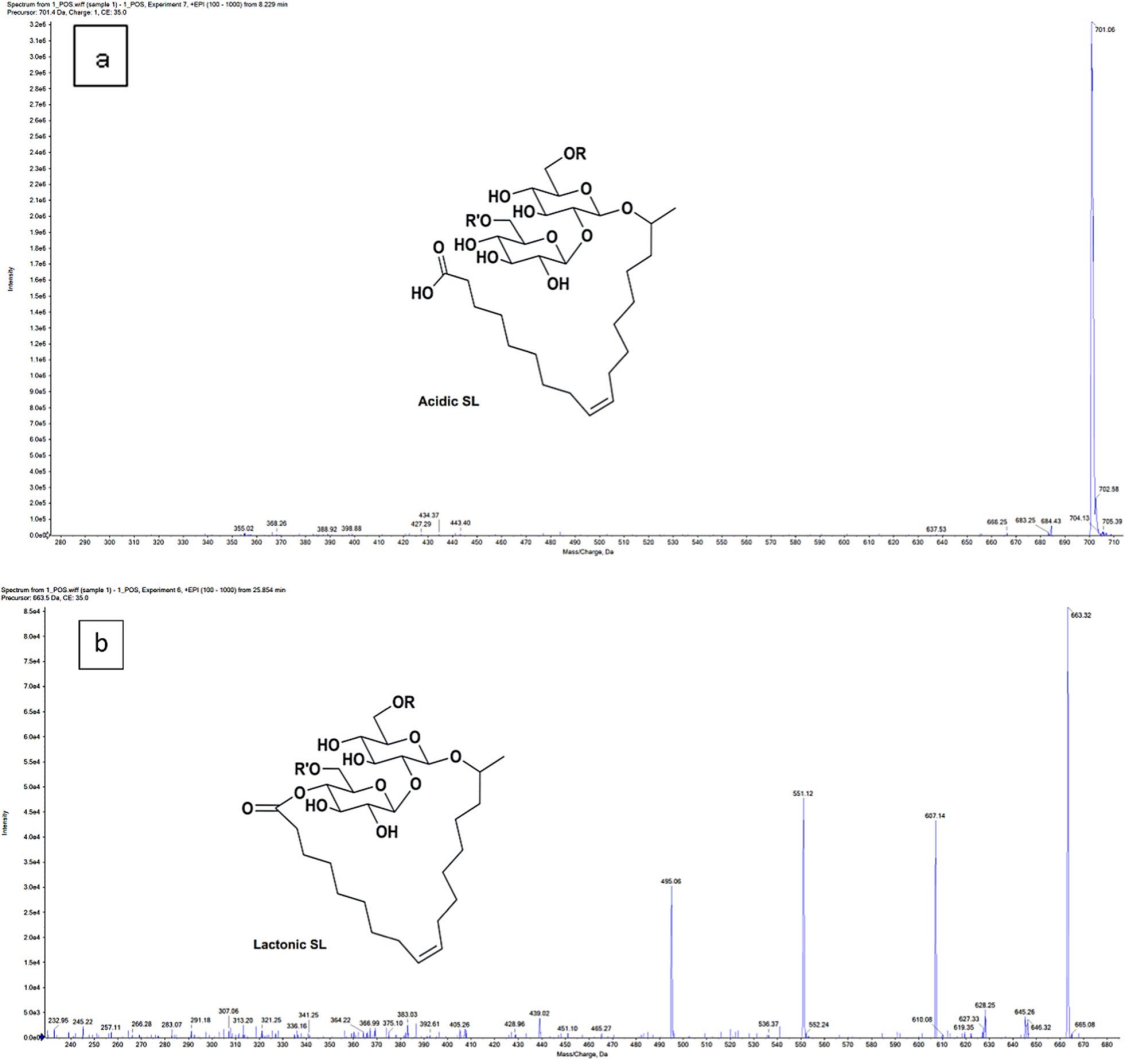
**a, b** Characterization of the SLs produced by *C. parapsilosis* using LC–MS in positive electrospray ionization mode (+ ESI). **a** The ion at m/z 701.31 corresponds to acidic SL at the retention time of 8.3 min and its fragments. **b** The ion at m/z 663.3 corresponds to lactonic SL at the retention time of 25.85 min and its fragments.

LC–MS analysis for the produced SLs was implemented according to the previously mentioned conditions. The results shown in (Fig. 7a, b) illustrated the presence of acidic and lactonic forms of SLs in the produced extract. The acidic SL was detected in the peak of a mass to charge ratio (m/z) 701.06 [9, 13], at the retention time of 8.23 min. The peak at m/z 443.4 was observed in the same retention time which corresponding to sophorose with fatty acid [13]. While the hydroxy fatty acid peak was observed at m/z 398.88.

The existence of lactonic form of SLs was indicated by the mass to charge ratio of 663.32 at the retention time of 25.85 min [38]. The peak observed at m/z 536.37 was correspond to the sophorose moiety with C15:3 fatty acid, and the peak at m/z 495.06 was related to the sophorose combined to C13 fatty acid [13]. While, the peak at m/z 405.26 indicated the presence of sophorose moiety [39]. However, at the same retention time, the hydroxy fatty acids noticed at 383.03 and 321.25 and fatty acid fragments were also spotted at m/z 283.07 and 257.11[13]**.**

### Emulsification activity of the produced SLs

**Fig. 8:**
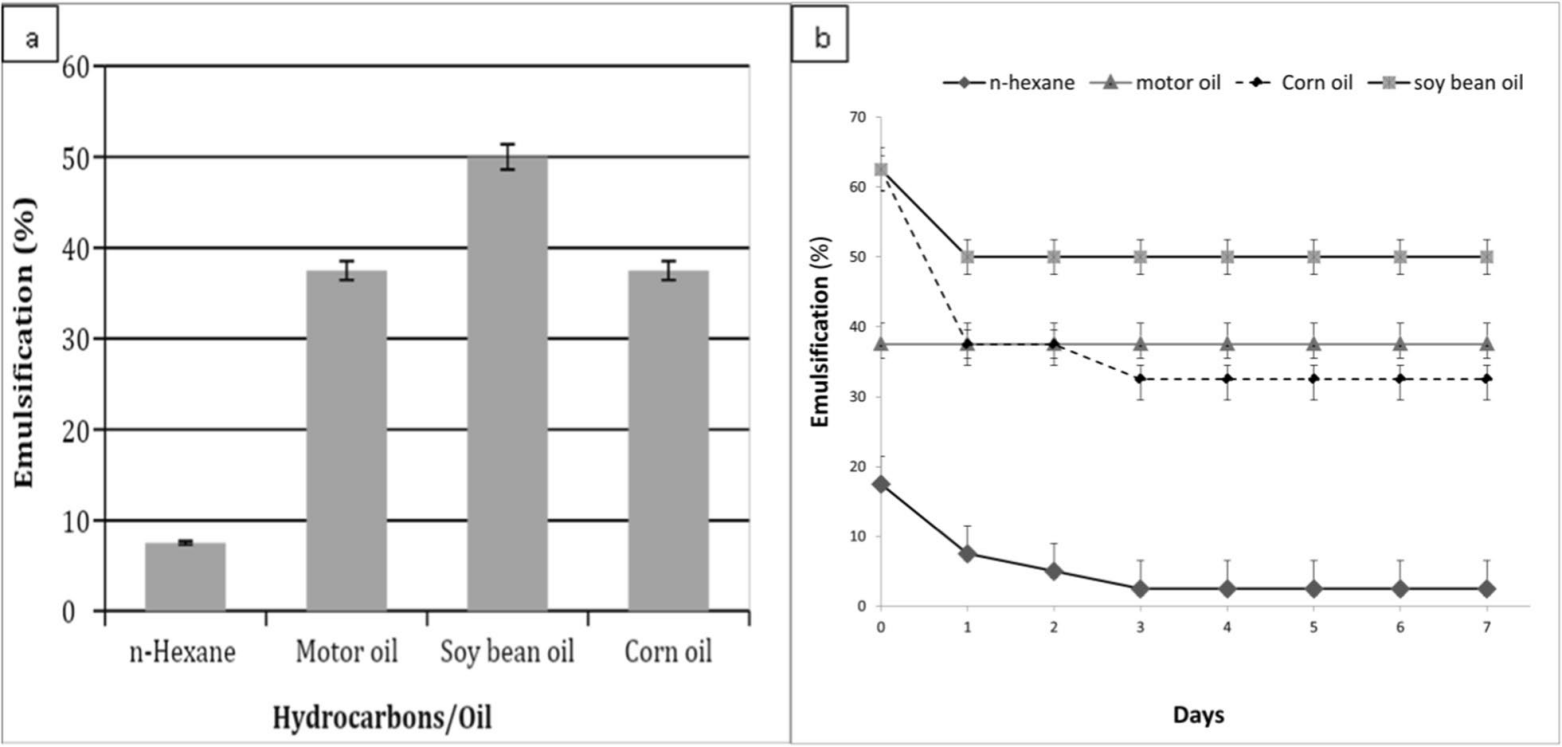
Efficiency of emulsifying activity toward different long and short hydrocarbons (**a**) and emulsification stability with soybean oil of the produced SLs (**b**) during 7 days at room temperature. Data were expressed as mean ± S.E. of 3 experiments

Stabilization of an oil and water emulsion is one of the important factors that determine the SLs potential applications at the industrial level. The emulsifying activity of the isolated SL with various short and long-chain hydrocarbon substrates at 25 °C is represented in (Fig. 8a). The results indicated the high emulsification activities of the produced SLs, specially towards soybean oil (E_24_ = 50%), followed by corn and motor oils (E_24_ = 37.5%). In contrary, short-chain hydrocarbon substrates namely n-hexane exerted the lowest emulsion values (E_24_ = 7.5%). These results are in fact illustrated the effectiveness of the produced SLs in forming stable emulsions with long-chain hydrocarbon substrates suggesting its suitability use in the industrial applications such as foods and drugs industry. [33], obtained lower emulsification index (E_24_ = 33.33%) for the vegetable oil (sunflower oil), from the biosurfactant produced by *Rhodotorula babjevae*, while the biosurfactant form *Cunninghamella echinulate* exhibited a similar index value about 60% for the soybean oil [40]**.** Recently, [34] tested vegetable oil (olive oil) which had a comparable index value for soybean oil. The results obtained for the motor oil emulsification index (E_24_ = 37.5%) was lower than *C. bombicola* SLs in which they recorded 60.7 and 46.9% for used and unconsumed motor oil, respectively [41].

### Emulsification stability studies of the produced SLs

Emulsification stability of the isolated methanol SLs extract with various short and long-chain hydrocarbon substrates during 7 days are shown in (Fig. 8b). The stability of emulsions with long chain hydrocarbons substrates (vegetable oils and motor oil) was more favorable than short chain ones. Stability results obtained were in agreement with the emulsification stability conducted on *Candida bombicola* [5] where they reported that the produced SL formed stable emulsions with the long chain hydrocarbons, however, they observed that the motor oil retained more than 50% of its activity during the 7 days of experiment, followed by soybean oil. Earlier, [42] mentioned the same results reported by [5] concerning the behaver of their produced SLs isolated from *C. guilliermondii.* In contrast to previous reports, the SLs produced in this study has a higher affinity towards vegetable oils compared to mineral one. Which actually makes it more suitable in applications related to the food industry.

### Environmental tolerance for the produced SLs

From the obtained results concerning environmental tolerance or industrial factors such as pH, salinity and temperature, it was found that environmental factors are affecting the emulsification activities significantly. Thus, this study is important, as it may limit or extend the future of utilizing biosurfactants for different applications.

**Fig. 9:**
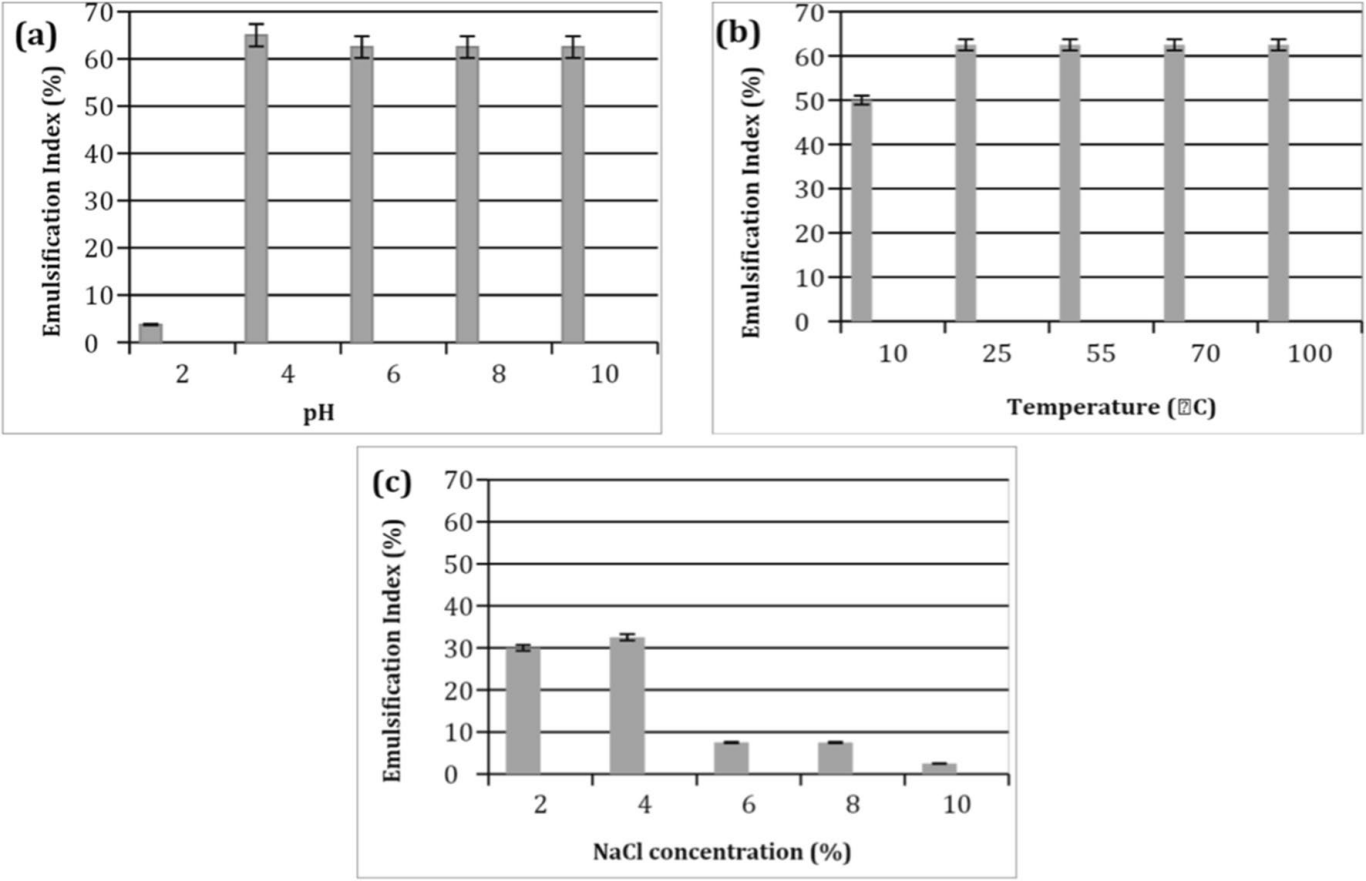
Environmental tolerance for the produced SL, presented by Heat stability (**a**), influence of pH (**b**) and the effect of NaCI concentrations (**c**) on the emulsifying activity towards soybean oil. Data were expressed as mean ± S.E. of 3 experiments

The emulsification index values of the methanol extracted SLs were measured for these factors (Fig. 9a, b and c). Regarding the effect of pH on the emulsifying activity, it was observed that the formed emulsions maintained their stability (62.5–65% activity) at broad pH values (4–10). From this it can be concluded that the produced SLs had a high tolerance in acidic and alkaline environmental conditions.

The results also, revealed that emulsification activities were stable against thermal treatment from 10 to 100 °C (50–62.5% activity). Whereas, the higher emulsification values were noticed from 25 °C to extreme temperature at 100 °C (62.5%), suggesting the tendency of the produced SLs to be applied in industrial high temperature systems (Fig. 9b). On the other side, the results in Fig. 9c, indicated the unsuitability of the obtained SLs emulsions to be employed in the saline solutions, where it kept only about 30% of its activity at 2 and 4% of salt concentrations, and the emulsions were collapsed at higher concentrations.

Almeida et al., [43] found that the SL produced by *C. tropicalis* emulsion with soybean oil was stable at a range of 30% but the activity was increased to about 70% in alkaline environment at pH 12. On the other hand, SL emulsification index levels were fluctuating when exposed to high temperatures from 70 to 120 °C. They also, reported higher level of emulsification stability with soybean oil against the salt concentration from 2 to 12%. Consequently, our results revealed that the produced SLs were able to withstand harsh environmental conditions concerning pH and temperature, allowing their suitability in many applications.

### Anti-Mucorales activity of SL

**Fig. 10:**
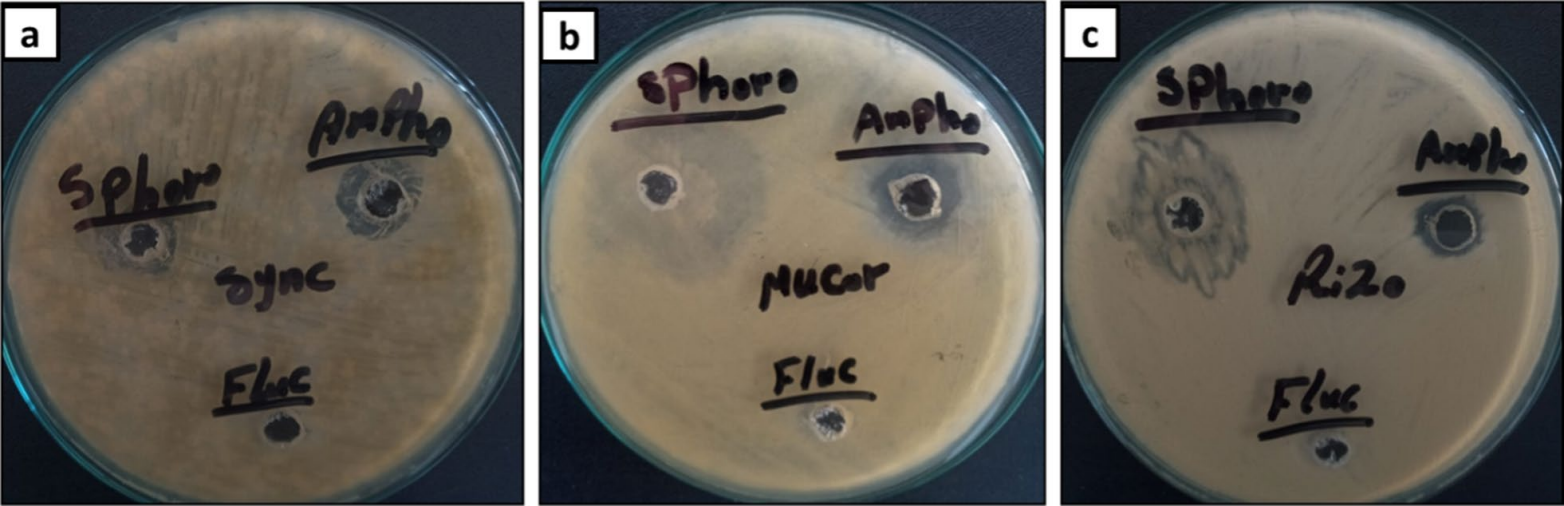
Anti-Mucorales activity of SL against *Syncephalastrum racemosum* (**a**), *Mucor racemosus* (**b**), and *Rhizopus microsporus* (**c**) as compared to standard drug: Fluconazole, and Amphotericin B

The efficacy of SL to serve as antifungal against major Mucorales species such as *Mucor racemosus, Rhizopus microsporus,* and *Syncephalastrum racemosum* was initially studied by agar-well method. The ability of methanol SL extract to prevent the proliferation of the Mucorales strains was evaluated at 100 µg/ well. As shown in (Table 4**, **Fig. 10), SL was found to have a significant anti-Mucorales activity towards all tested pathogens with a diameter of the inhibition zones varying from 5 to 9 mm. On contrast, there was no any antifungal activity of Fluconazole against all Mucorales strains, since all of the tested organisms showed high resistance even at 400 µg/ well of fluconazole, while Amphotericin B had an inhibition activity at 40 µg/ well against all tested strains with inhibition zones between 3 and 5 mm.

**Table 4:** Anti-Mucorales activity of the SL

Sample	*Inhibition Zone (mm)*		
	*Rhizopus microsporous*	*Syncephalastrum racemosum*	*Mucor racemosus*
SL	7 ± 0.05	4 ± 0.22	9 ± 0.02
Fluconazole^a^	ND	ND	ND
Amphotericin B	5 ± 0.11	4 ± 0.18	3 ± 0.05

*SL* sample was used at 100 µg/well concentration, *ND* not determined

^a^Antifungal agent (Positive control) at 40 µg/well concentration

It is noteworthy that SL showed the most outstanding inhibitory effect against *M. racemosus*, and *R. microsporus* with 9 ± 0.02 mm, and 7 ± 0.05 mm respectively which was more than inhibition activity of Amphotericin B (Fig. 10). On contrast, *S. racemosum* had an inhibition activity less than the standard drug Amphotericin B. Subsequently, SL with significant antifungal activity was utilized for further experiments in order to determine MIC at the concentrations ranging 25–400 µg/ml.

Although the MIC values of SL against the tested fungal strains are high in compared to the standard drug (Table 5), the promising inhibition results of SL was opining a way to apply this SL in the pharmaceutical industries in order to promote the killing effect of the standard drug such as Amphotericin B, and posaconazole.

**Table 5:** Minimum inhibitory concentration (MIC) of SL against Mucorales strains

Sample	Minimum Inhibitory Concentration (MIC, µg/ml)		
	*Rhizopus microsporus*	*Syncephalastrum racemosum*	*Mucor racemosus*
SL	250 ± 4.25	125 ± 3.33	100 ± 6.15
Amphotericin B	31.5 ± 0.88	62.5 ± 2.55	62.5 ± 4.82

In accordance with our results, SL has broad spectrum of antimicrobial activity toward numerous bacterial and fungal pathogens [28, 44]. The obtained results showed that, the *C. parapsilosis* SL displayed a distinguished disruptive susceptibility on fungal mycelial growth and prevent the conidial germination. In deeply, the conidia germination in fungal cells attached with the epithelial cells for germination and thus initiate an invasion of the stratum corneum layer. The infection of Mucorals strains is established by the penetration of hyphae to the epidermis cells and the infections in the outer layers of the tissues are disrupted at regular intervals [45]. Anti-fungal activity of SL was observed dependent on the types of fatty acid structure, in which the SL composed of several lactonic rings demonstrated highest antimicrobial activity as compared to the acidic SL form [46]. Antifungal activity of SL against the foodborne fungi was reported by [12, 46]**,** which was success in controlling the fungal proliferation and enhancing the food preservations. Particularly for people with impaired immune systems, infections with opportunistic fungus, such as those from the Mucorales, are becoming more prevalent [22]. The classical treatment of mucormycosis with Amphotericin B, posaconazole and isavuconazole are recently facing some problems such as increasing resistance by some Mucorales strains, and the severe side effects of these drugs under high concentrations [22]. Therefore, new strategies bases on the preparing of modified drugs, are in persistent need to solve these hinders such as combination of the potent anti-mucormycosis agent (Amphotericin B) with another natural compounds. This combination may reduce the high drug concentration used for the treatment and consequently its toxicity, moreover it may provide a synergistic effect against Mucorales strains. Furthermore, this combination may be useful in transferring some of the characteristics of SL compounds, such as the ability to form a stable emulsion tolerant for a wide range of temperature and pH, which improves the properties of the drug.

## Conclusion

*Candida parapsilosis* was found to be the most robust strain to produce SLs among the tested organisms that were isolated from an area of chemical industries. The production including optimization process improved the yield and the ST of the produced SLs by 30 and 23%, respectively. Emulsification studies, on the other hand proved the affinity of the produced SLs to vegetable oils as well as mineral oils with high tolerance against extreme environmental conditions such as pH and temperature, suggesting their potential applications in different industries. It is also clear that the produced SLs have a high ability to inhibit the growth of fungi causing mucormycosis, as it was given excellent inhibition efficiency against all Mucorales strains. To our knowledge this is the first study concerning the SLs activity against Mucorales strains that capable to rapid proliferation in the immunocompromised patients. Generally, this may establish a new trend in pharmaceuticals for the emergence of a new generation of effective and safer treatments for black fungi, especially in light of the spread of the Coronavirus and its impact on the public health.
